# Supplementary material for: The HD-ZIP II Transcription Factors HAT3 and ATHB4 Fine-Tune Auxin and Cytokinin Pathways During Flower Development
Source: Plants (Basel). 2025 Dec 6;14(24):3723. doi: 10.3390/plants14243723 (PMC12736722; doi:10.3390/plants14243723)
Supplement: Supplementary file 1 [file plants-14-03723-s001.zip › plants-3980405-supplementary.pdf]

Supplementary Materials

The HD-ZIP II Transcription Factors HAT3 and ATHB4 Fine-Tune Auxin and Cytokinin Pathways during Flower Development

Kestrel A. Maio[1], Sophia Luche[2], Monica Carabelli[3] and Laila Moubayidin[2,*]

1: John Innes Centre, Norwich Research Park, Colney Ln, Norwich NR4 7UH, United Kingdom.

2: Dipartimento di Scienze Chimiche, della Vita e della Sostenibilità Ambientale, University of Parma, Parco Area delle Scienze 11/A, 43124 Parma, Italy.

3: Institute of Molecular Biology and Pathology, National Research Council, P.le A. Moro 5, 00185 Roma, Italy.

* Corresponding author: laila.moubayidin@unipr.it

Supplemental Table S1. Gene Ontology (GO) categories associated with genes predicted to be functionally related to HAT3 and ATHB-4 following GeneMANIA network analyses.

GO ID	GO Annotation	Coverage	Q-value	Genes
GO:0045595	regulation of cell differentiation	4/29	2.50E-04	HDG1, HDG2, HDG12, GL2,
GO:0090558	plant epidermis development	5/225	0.015	HDG2, HDG11, HDG12, ANL2, GL2
GO:0051093	negative regulation of developmental process	4/127	0.034	HDG1, HDG2, HDG12, GL2
GO:0010026	trichome differentiation	3/65	0.151	HDG2, HDG11, HDG12
GO:0090626	plant epidermis morphogenesis	3/78	0.209	HDG2, HDG11, HDG12
GO:0030855	epithelial cell differentiation	2/17	0.311	ATML1, GL2
GO:0008544	epidermis development	2/17	0.311	ATML1, GL2
GO:0048468	cell development	4/286	0.311	HDG2, HDG11, HDG12, ANL2
GO:0060429	epithelium development	2/20	0.380	ATML1, GL2
GO:0010090	trichome morphogenesis	2/32	0.887	HDG11, HDG12

Supplemental Table S3. List of primers used in the present study.

Oligo Name	Sequence (5'->3')	Reference
ACT2-f	AATCACAGCACTTGCACC	[68]
ACT2-r	ATTCCTGGACCTGCCTC	[68]
AMI1-f	GCTCAGAGCTTCGACACAG	[69]
AMI1-r	CCGTCTGAGCCATACTTAGC	[69]
CKX3-f	AGCGGTCCTGTTCTTGTTTATCCT	[70]
CKX3-r	ACCTCGGACCAAAATGTCTAACCC	[70]
CYP79B2-f	TGACGGATCCCAACAAAAAG	[71]
CYP79B2-r	ATGATCGGCCATCCTGTG	[71]
PUP14-f	CGAGCGTGTTGTCGAATGTG	[26]
PUP14-r	GGCAGTAACCAAGGCAACAC	[26]
SUR1-f	CAGGCATATCTAAGGGATGGGTTG	[71]
SUR1-r	AATTATTGTGGCAGGGTCAGGAG	[71]
UB10-f	GGCCTTGTATAATCCCTGATGAATAAG	[72]
UB10-r	AAAGAGATAACAGGAACGGAAACATAGT	[72]
YUC3-f	CGTCCCTTCATGGCTTAAGGACAAC	[73]
YUC3-r	GACGCACCAAACAATCCTTTTCTCG	[73]
YUC5-f	ATGATGTTGATGAAGTGGTTTCCTCTG	[73]
YUC5-r	ATCAGCCATGCAAGAATCAGTAGAATC	[73]
YUC6-f	GAGACGCTGTGCACGTCCTA	[73]
YUC6-r	AGTATCCCCGAGGATGAACC	[73]
YUC8-f	ATCAACCCTAAGTTCAACGAGTG	[73]
YUC8-r	CTCCCGTAGCCACCACAAG	[73]

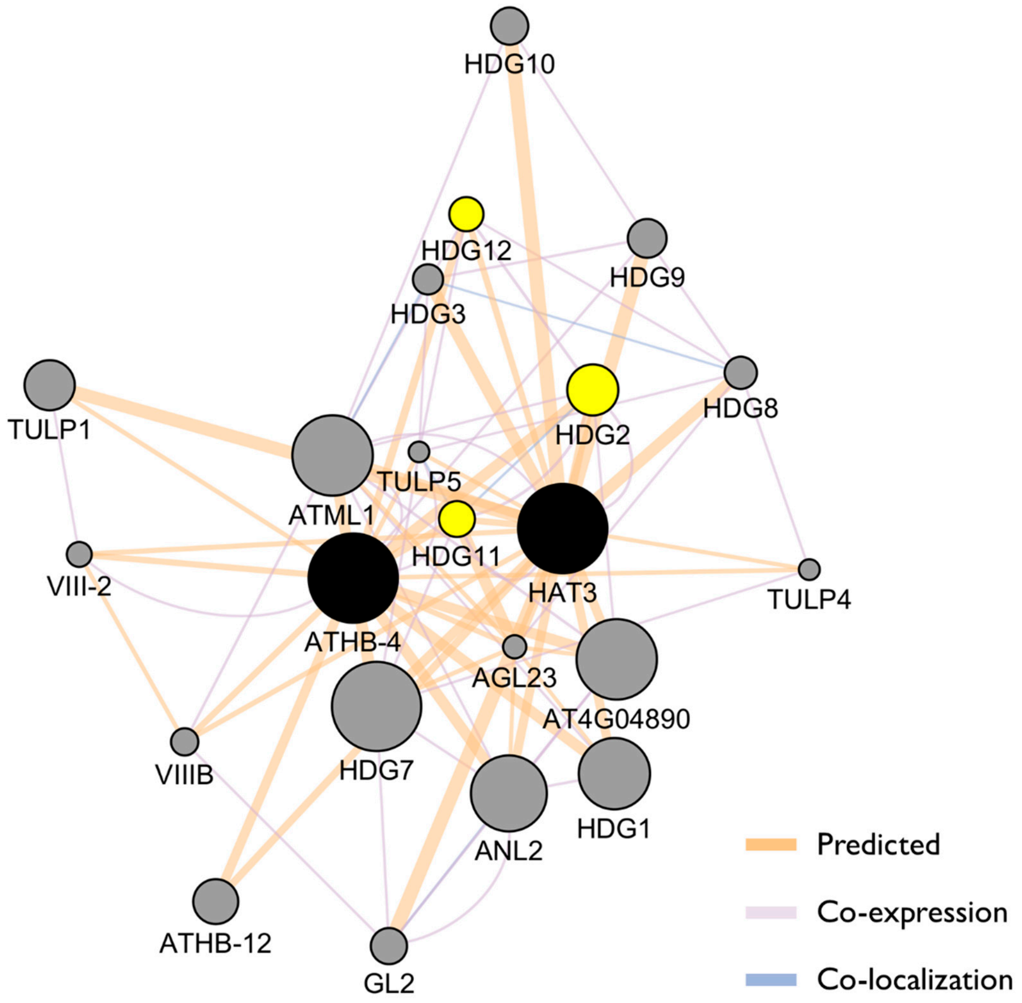

Supplemental Figure S1. Gene-gene interaction network of the HAT3 and ATHB4 transcription factors. Data were obtained from GeneMANIA Cytoscape plugin. Query genes are represented by black circle, while functionally related genes are represented by grey circles. Genes involved in the GO category “Trichome differentiation” are highlighted in yellow. Edges highlighting interactions based on prediction, co-expression, or co-localization are shown in orange, pink, and blue, respectively. The thickness of each edge is proportional to the weight of the interaction.
